# Racial Differences in Patient-Reported Post-Stroke Disability in Older Adults

**DOI:** 10.3390/geriatrics2020016

**Published:** 2017-05-23

**Authors:** Charles Ellis, Gayenell Magwood, Brandi M. White

**Affiliations:** 1 Department of Communication Sciences & Disorders, College of Allied Health Sciences, East Carolina University, 3310H Health Sciences Building, MS 668, Greenville, NC 27834, USA; 2 College of Nursing, Medical University of South Carolina, 97 Jonathan Lucas Ave, Charleston, SC 29425, USA; magwoodg@musc.edu; 3 Division of Healthcare Studies, College of Health Professions, Medical University of South Carolina, 151-B Rutledge Ave, Charleston, SC 29425, USA; whitbm@musc.edu

**Keywords:** stroke, race, disparities, outcomes

## Abstract

Longstanding disparities have been reported in stroke-related outcomes with blacks experiencing more post-stroke disabilities. Little is known about long-term disability outcomes among older stroke survivors. This study was a retrospective analysis of data from the 2015 National Health Interview Survey (NHIS). A group of 655 stroke survivors (541 white and 114 black) age 65 and older were asked to rate their ability to complete 10 functional tasks without special equipment. Univariate comparisons were completed using *t*-tests and chi-square statistics for racial comparisons of disability reports. Multinomial logistic regression was used to determine odds of reporting disability after controlling for relevant covariates. The mean age of the sample was 76.6 years. After controlling for relevant covariates, white stroke survivors were less likely to report the following tasks being “very difficult/can’t do at all” without using special equipment compared to blacks: reach overhead (OR = 0.39, 95% CI 0.23–0.65; *p* = 0.000) and grasp small objects (OR = 0.42, 95% CI 0.25–0.73; *p* = 0.002). Both black and white older stroke survivors experience significant post-stroke disability across a range of functional tasks. Slightly greater long term post-stroke disability appears to exist among older blacks.

## 1. Introduction

The worldwide burden of stroke is substantial as approximately 15 million individuals experience a stroke annually [1]. In the United States (US) alone, it is believed that 795,000 Americans experience a stroke each year [2]. A significant racial disparity in stroke exists as Non-Hispanic Blacks (Blacks) are at twice the risk of having a stroke for the first time compared to Non-Hispanic Whites (Whites) [2,3,4]. Greater stroke risk persists in the US among Blacks despite an overall decrease in ischemic stroke incidence among Whites in the US [5].

A secondary concern in the study of stroke-related racial disparities is that Blacks are more likely to experience strokes that culminate in greater stroke-related limitations [6]. To date, studies related to racial differences following the completion of rehabilitation have been mixed with some studies reporting worse outcomes among Blacks and others reporting no significant differences [6,7,8,9]. For example, a systematic review of post-stroke outcomes after rehabilitation that included 17 studies and 429,108 stroke survivors showed that Black stroke survivors were generally more likely to achieve poorer functional outcomes than White stroke survivors [10].

Currently, there is an expansive literature related to racial disparities in stroke outcomes. Yet, few studies have emphasized the study of older stroke survivors (>age 65). Although, three-quarters of individuals who experience a stroke are over that age of 65 [2], less attention has been given to the influence of race on outcomes in the older stroke population. Because the stroke population is heterogeneous in regards to age and other sociodemographic characteristics, studies of racial disparities in stroke-related outcomes may benefit from studying patients population groups that are similar in age. Additionally, Blacks and other racial-ethnic minorities are more likely to experience strokes at a younger age [2] and traditional age adjustments may not reflect actual functional performance. A review of the current literature related to racial/ethnic differences in stroke-related outcomes among older adults yielded three studies related to stroke risk, mortality, and quality of care. Yet, none offered information about functional outcomes or functional performance. See Table 1 for a summary of studies reporting racial differences in post-stroke outcomes among older adults.

The source of the observed mixed results across studies of stroke is unclear. Some have suggested that measurement approaches, timing of measurement during the recovery process, and other unmeasured contributors to outcomes are key contributors to the observed racial disparities [14]. In addition to measurement issues, less attention has been given to patient-reported outcomes (PROs) that capture the stroke survivor’s perspective of the recovery process and their abilities. Patient reports of disability or functional performance offers critical information regarding the personal and interpersonal impact of conditions such as stroke [15]. Additionally, obtaining PROs provides the best understanding patient performance from their perspective and how their performance impacts them in their particular context, i.e., circumstances.

Therefore, the purpose of this study was two-fold. First, to measure the “functional capacity” or ability to complete functional tasks independently or with an assistive device and compare those reports between racial groups of older stroke survivors (≥65 years old). Although stroke is primarily a condition of older adults, less is known about the functional ability of the oldest stroke survivors or whether racial differences in outcomes are present among them [2]. Second, to complete and emphasize patient-reported measures (outcomes) to explore older stroke survivor’s perspective of their functional abilities or level of disability. To complete this study, data from the 2015 National Healthcare Interview Survey (NHIS) were utilized [16]. The NHIS can be utilized to complete a cross-sectional examination of racial differences in the functional performance among stroke survivors.

## 2. Materials and Methods 

### 2.1. Data Source

Data from the 2015 NHIS were utilized for this study. The NHIS is an annual cross-sectional household survey of non-institutionalized adults over the age of 18 administered by the US Bureau of the Census for the National Center for Health Statistics (NCHS) [16]. The primary objective of the NHIS is to evaluate and monitor the health of the United States population on a range of health-related topics. The survey examines use of healthcare services, health behavior, and general health status. The study was reviewed and approved the East Carolina University (ECU) institutional review board (IRB).

### 2.2. Study Population

The study population consisted of adult respondents who self-reported a history of stroke. All respondents answered “yes” to the following question: “Have you ever been told you had a stroke?”

### 2.3. Demographic Characteristics and Co-Morbid Disease

Key variables included: race, sex (male vs. female), stroke duration in years, and age in years. Race was limited to non-Hispanic Black (Blacks) and Non-Hispanic White (Whites) due to limited representation of other race and ethnicity categories. Age was treated as a continuous variable and categorical variable using the following age categories: 65–74, 75–84, and >85. Marital status was classified as: married, widowed, and divorced/separated/other. Presence of the following comorbid conditions was identified and based on self-report: hypertension, high cholesterol, heart disease, angina, heart attack, chronic obstructive pulmonary disease (COPD), and diabetes.

### 2.4. Primary Outcome Variable

The primary outcome variable was a self-report of physical capacity based on responses to the following: “How difficult is it to […………] without special equipment (walk a quarter mile, climb 10 steps, stand 2 h, sit 2 h, stoop, reach overhead, grasp small objectives, carry 10 pounds, push large objects, go to events, and participate in social activities)?” Response categories included: not difficult at all, only a little difficult, somewhat difficult, very difficult, can’t do at all, or do not do this activity. For analysis purposes, the responses were reclassified as: not at all, little difficult/somewhat difficult, very difficult/can’t do at all, or do not do this activity.

### 2.5. Data Analysis

Data analyses were completed using IBM SPSS Statistics 22 [17]. Descriptive statistics were completed to describe the total sample. For baseline race comparisons, *t*-tests were used for continuous variables (age and stroke duration) and Chi-square statistics for categorical variables (age categories, sex, and disease conditions). Individual Chi-square statistics were also used for univariate comparisons of the 10 disability outcome measures. Multinomial logistic regression was used to obtain the odds ratio of reporting “little difficult/somewhat difficult” and “very difficult/can’t do at all” using the “not at all” as the reference category. The 10 individual multinomial logistic models were controlled for any observed baseline racial differences in demographic characteristics (e.g., age) or comorbid diseases (e.g., hypertension). Odds ratios (OR) and 95% confidence intervals were calculated and reported for each regression model.

## 3. Results

The 2015 NHIS included 1078 adults with a history of prior stroke. Approximately 60% of the sample was ≥75 years old, 57.1% were women and 82.6% were White. The mean age for the sample was 76.7 (SD ± 7.0) years and the mean years post-stroke was 10.3 (SD ± 15.9) years. Thirty percent of the sample was married and 44% were widowed. Approximately 78% of the sample reported having hypertension, 67% high cholesterol, 28% heart disease, 15% angina, 24% heart attack, 17% COPD, and 34% diabetes. There were significant racial-ethnic differences by marital status, sex, and presence of diabetes. Table 2 reports total sample demographic and comorbid disease characteristics and comparison by race.

### 3.1. Racial Differences in Disability

In univariate comparisons, statistically significant racial differences were reported in difficulty being able to complete the following tasks without special equipment: sitting for 2 h, reaching overhead, grasping small objects, and participating in social activities. White stroke survivors were less likely to report “very difficult/can’t do at all” in sitting for 2 h (9% vs. 13%; *p* = 0.045), reaching overhead (12% vs. 26%; *p* = 0.000), and grasping small objects (12% vs. 24%; *p* = 0.000). Similarly, Whites were less likely to report “little difficult/some difficult participating in social activities (12% vs. 21%; *p* = 0.021) when compared to Blacks. No racial differences were observed in walking a quarter mile, climbing 10 steps, standing for 2 h, stooping, carrying 10 pounds, pushing large objects and going to events. See Table 3 for a summary of physical capacity by race.

### 3.2. Odds of Disability by Race

Table 4 shows the odds of reporting difficulty being able to complete the 10 tasks without using special equipment. With “not at all” as the reference category and adjusting for sex and presence of diabetes, Whites were less likely to report “very difficult/can’t do at all” in reaching overhead (OR = 0.39, 95% CI 0.23–0.65; *p* = 0.000) and grasping small objects (OR = 0.43, 95% CI 0.25–0.73; *p* = 0.002). Although the focus was on the more disabling category (very difficult/can’t do at all), Whites were also less likely to report “little difficult/somewhat difficult” participating in social activities (OR = 0.45, 95% CI 0.26–0.80; *p* = 0.000) when compared to Blacks.

## 4. Discussion

Stroke is a condition that can result in substantial physical disability thereby limiting the stroke survivor’s ability to engage in functional daily tasks. The key finding of this study was that older White stroke survivors are less likely to report measures of physical capacity being “very difficult” or they had an “inability to complete” the tasks when compared to Blacks. These findings support previous reports of greater disability among Black stroke survivors [2,18,19]. More specifically, data from the 2000–2001 NHIS [18] and those of Ellis and colleagues [19] both found greater functional limitations among Black stroke survivors. Additionally, the findings reported here show a persistence in racial disparities in post-stroke disability using the same patient-reported outcomes from the NHIS 14 years later but with a primary focus on older adults. These findings offer additional insights as studies of racial disparities in stroke-related outcomes are traditionally completed at discharge or within six months of discharge. The data reported here are from community-based older stroke survivors with multiple years post-stroke onset.

These findings highlight the complex combination of disabling levels of functional disability that many stroke survivors experience. Additionally, if older Blacks experience greater disability among more physically intense tasks than older Whites, they are more likely to experience greater limitations in the home and community settings. Greater disability among Blacks agrees with previously published data from the 2011 National Health and Aging Trends Study which also showed that Black stroke survivors were more likely to report limitations in self-care, mobility, and household activities when compared to White stroke survivors [20]. The authors concluded that physical capacity limitations (being able to complete the task independently or with an assistive device) accounted for the majority of racial disparities observed in the study. However, the sample consisted of young and old stroke survivors. Consequently, including a sample that is heterogeneous in age does not facilitate a clear understanding the impact of reported on how that disability impact individuals of different ages across the racial-ethnic groups.

The findings here are critically important because they offer additional evidence of long-term racial disparities in stroke-related outcomes among older adults. Previous studies have offered conflicting evidence regarding race and recovery trajectories over time. For example, Bhandari and colleagues found that Blacks achieved less functional improvement after discharge from community rehabilitation when compared to Whites [6]. Similarly, Putman and colleagues found less functional improvement among Blacks compared to Whites despite Blacks having high functional scores on admission [21]. In contrast, Horn et al. and Deutscher and colleagues found no significant racial differences in outcomes following stroke rehabilitation [8,9]. Therefore, less is known about the long-term implications of stroke recovery and potential variations between racial groups or how age older age influences observed disparities.

In addition, the findings here offer information that emphasizes only the patient’s perspective to the stroke survivor’s functional abilities. The study of stroke outcomes and specifically racial disparities in outcomes has been confounded by a number of factors associated with outcome measurement. Varying measurement approaches, timing of measures, and settings where measurements are traditionally completed have been proposed as a critical reason why reports in disparities in outcomes have been inconsistent [19]. According to Barak and Duncan, because measurement varies in the domain assessed (impairment, functional performance, etc.), approach (performance based, patient based, etc.), and timing (hospital discharge, rehab discharge, etc.), the sensitivity of such measures may not be equivalent and or designed to capture racial disparities in outcomes [14]. Therefore, the findings reported here only relate to the stroke survivor’s perception of the ability to complete the specified tasks. Consequently, these findings should only be compared to studies with the same or similar tasks and solely based on patient report. These findings also suggest stroke-related functional disability persists over a longer period of time, into old age and likely the lifespan of stroke survivors.

Although older Blacks reported greater post-stroke disability, it is important to acknowledge that explanations of racial disparities in stroke outcomes are not straight forward. According to Burke and colleagues, measures of physical capacity such as those used in this study can be associated with pre-stroke capacity, stroke type and severity, stroke-related care, use of rehabilitation and other factors in the post-stroke environment [20]. Because the stroke survivors in this study were all community stroke survivors, it is extremely important to note the absence of critical information about post-stroke social support. Although there were racial differences in marital status, a better metric of the support available to the stroke survivor likely community support. The spouses of many older stroke survivors also have chronic diseases or disabling conditions that limit their support of the older stroke survivor. Hinjosa and colleagues found that social support and the network of individuals involved in the stroke care process can be critical to optimal stroke recovery [22]. Additionally, they found that these “stroke social support networks” can vary by race-ethnicity.

The impact of stroke social/community support networks must be carefully considered in this study and all studies of racial disparities in stroke outcomes. The presence of strong and diverse stroke social support networks prior to sustaining a stroke has been shown to be associated with more positive stroke outcomes [23]. Beyond physical assistance, the contributions of these networks are not entirely clear. In addition, it is possible that race-based cultural differences may influence stroke survivor vs. support network expectations for such activities as activities of daily living and driving [6]. Therefore, future studies of disparities in stroke outcomes and in particular measures of long-term outcomes should give careful consideration to the support stroke survivors receive during the recovery process.

Although the findings reported here are interesting, this study is not without limitations. First, post-stroke duration was not available for all stroke survivors. Post-stroke duration was available for less than 25% of the total sample (15% of Whites and 35% of Blacks). Second, patient reported outcomes can differ substantially from those of healthcare providers and therefore it may be expected that differences also exist among patients with similar disability profiles [24]. Third, although the Black stroke survivors represented >17% of the sample, a more equivalent sample of Black stroke survivors may have offered different results. Fourth, controlling for co-morbid diseases such as diabetes may not adequately capture the complex nature of multiple chronic disease on overall stroke recovery. This is of even greater importance when considering the impact in older adults who have experienced the disease conditions for multiple decades. Fifth, the survey does not collect data on the number of strokes the respondent has experienced. Sixth, the information related to initial stroke severity and need for or completion of stroke rehabilitation was not available. However, it is extremely important to note that, even in studies controlling for initial stroke severity, racial disparities exist in post-stroke disability [19]. Seventh, the data reported here was based on self-report which can be influenced by declines in memory and other cognitive skills. Therefore, the relevance of initial stroke severity in relationship to functional ability is not entirely clear when studying racial disparities in outcomes.

## 5. Conclusions

Despite the aforementioned limitations, the findings reported here offer significant evidence of significant post-stroke disability in older adults with some racial disparities in stroke-related functional ability. However, additional studies need to adequately explain the complex range of patient and likely environmental factors that contribute to functional limitations in older stroke survivors and the observed racial disparities in this study.

## Figures and Tables

**Table 1:** Studies examining racial differences in post-stroke outcomes among older stroke survivors.

Author	Year	Sample	Outcome	Results
Qian et al. [11]	2013	Black, White, Hispanic, Asian patients from US Centers participating in Get with Guidelines Program	Mortality	Asians had lower 30 day and 1 year mortality compared to all other groups after risk adjustment for stroke severity and other prognostic variables
Li et al. [12]	2016	Black, White, Hispanic, Asian, Other patients from US Centers participating in Get with Guidelines Program-Coronary Artery Disease Registry	Risk factors Morality Quality of Care	Blacks had higher diabetes, hypertension, dialysis, and mortality but similar in measures of quality of care
Dupre [13]	2016	Black and White patients from Health and Retirement Study	Stroke risk factors Stroke risk	Blacks had higher risk of marital instability, risk factors, and stroke rates

**Table 2:** Demographic characteristics of older stroke survivors in 2015 national health interview survey.

	All	White	Black	*p*-Value
	n = 655	n = 541	n = 114	
**Stroke Duration, mean years (SD) (range <1 year–33 years)**	10.28 (15.9)	8.75 (14.57)	14.13 (18.68)	0.108
**Age, mean years (SD) (range 65–85)**	76.73 (6.99)	76.91 (6.59)	75.84 (7.13)	0.141
**Age (categories)**	%	%	%	0.498
65–74 years	39.8	38.8	44.7	
75–84 years	37.7	38.3	35.1	
≥85 years	22.4	22.9	20.2	
**Marital Status**				0.000
Married	29.8	32.9	14.9	
Widowed	44.0	43.3	47.4	
Divorced/Separated/Other	26.3	23.8	37.7	
**Sex: Female**	57.1	54.9	67.5	0.018
**Hypertension**	77.7	75.8	86.8	0.079
**Cholesterol**	67.3	67.8	64.9	0.830
**Heart Disease**	28.1	29.8	20.2	0.198
**Angina**	15.0	15.5	12.3	0.541
**Heart Attack**	24.4	25.5	19.3	0.294
**COPD**	16.6	17.9	10.5	0.073
**Diabetes**	33.7	30.3	50.0	0.001

**Table 3:** Reports of disability among older black and white stroke survivors.

How Difficult Is It to […………] without Special Equipment	All	White	Black	*p*-Value
	%	%	%	
**Walk 1/4 Mile**				0.858
Not at all	27.3	27.9	24.6	
Little difficult/Somewhat difficult	16.5	16.1	18.4	
Very difficult/Can’t do at all	43.5	43.3	44.7	
Do not do this activity	12.7	12.8	12.3	
**Climb 10 Steps**				0.250
Not at all	37.1	38.3	31.6	
Little difficult/Somewhat difficult	20.6	20.7	20.2	
Very difficult/Can’t do at all	35.6	35.1	37.7	
Do not do this activity	6.4	6.5	10.5	
**Stand 2 h**				0.946
Not at all	24.9	25.1	23.7	
Little difficult/Somewhat difficult	17.1	17.2	16.7	
Very difficult/Can’t do at all	48.4	47.9	50.9	
Do not do this activity	9.6	9.8	8.8	
**Sit 2 h**				0.045
Not at all	70.1	71.3	64.0	
Little difficult/Somewhat difficult	18.2	18.1	18.4	
Very difficult/Can’t do at all	10.1	9.4	13.2	
Do not do this activity	1.7	1.1	4.4	
**Stoop**				0.125
Not at all	24.9	25.1	23.7	
Little difficult/Somewhat difficult	22.1	21.4	25.4	
Very difficult/Can’t do at all	46.7	48.1	40.4	
Do not do this activity	6.0	5.0	10.5	
**Reach over head**				0.000
Not at all	63.5	66.9	47.4	
Little difficult/Somewhat difficult	18.9	18.5	21.1	
Very difficult/Can’t do at all	14.7	12.2	26.3	
Do not do this activity	2.9	2.4	5.3	
**Grasp small objects**				0.000
Not at all	62.0	63.8	53.5	
Little difficult/Somewhat difficult	23.5	24.4	19.3	
Very difficult/Can’t do at all	13.6	11.5	23.7	
Do not do this activity	.9	.4	3.5	
**Carry 10 pounds**				0.053
Not at all	45.3	47.5	35.1	
Little difficult/Somewhat difficult	19.8	20.0	19.3	
Very difficult/Can’t do at all	28.2	26.8	35.1	
Do not do this activity	6.4	5.5	10.5	
**Push large objects**				0.303
Not at all	34.2	35.7	27.2	
Little difficult/Somewhat difficult	18.8	19.0	17.5	
Very difficult/Can’t do at all	35.7	34.6	41.2	
Do not do this activity	11.0	10.4	14.0	
**Go to events**				0.142
Not at all	48.5	50.5	39.5	
Little difficult/Somewhat difficult	17.1	16.5	20.2	
Very difficult/Can’t do at all	25.6	25.1	28.1	
Do not do this activity	8.7	7.9	12.3	
**Participate in social activities**				0.021
Not at all	54.0	56.4	43.0	
Little difficult/Somewhat difficult	13.4	11.8	21.1	
Very difficult/Can’t do at all	22.3	22.0	23.7	
Do not do this activity	10.2	9.8	12.3	

**Table 4:** Odds of reports of disability by race.

How Difficult Is It to […………] without Special Equipment	OR (Whites)	95% CI	*p*-Value
**Walk 1/4 Mile**			
Little difficult/Somewhat difficult	0.755	0.400–1.425	0.386
Very difficult/Can’t do at all	0.935	0.559–1.562	0.796
**Climb 10 Steps**			
Little difficult/Somewhat difficult	0.902	0.505–1.611	0.728
Very difficult/ Can’t do at all	0.938	0.570–1.543	0.800
**Stand 2 h**			
Little difficult/Somewhat difficult	0.990	0.515–1.902	0.976
Very difficult/ Can’t do at all	0.964	0.579–1.605	0.888
**Sit 2 h**			
Little difficult/Somewhat difficult	0.897	0.522–1.543	0.696
Very difficult/ Can’t do at all	0.682	0.360–1.292	0.240
**Stoop**			
Little difficult/Somewhat difficult	0.814	0.450–1.470	0.494
Very difficult/ Can’t do at all	1.367	0.890–2.332	0.251
**Reach over head**			
Little difficult/Somewhat difficult	0.652	0.382–1.115	0.119
Very difficult/ Can’t do at all	0.385	0.227–0.654	0.000
**Grasp small objects**			
Little difficult/Somewhat difficult	1.089	0.639–1.856	0.754
Very difficult/ Can’t do at all	0.427	0.250–0.732	0.002
**Carry 10 pounds**			
Little difficult/Somewhat difficult	0.850	0.475–1.523	0.585
Very difficult/ Can’t do at all	0.636	0.387–1.045	0.074
**Push large objects**			
Little difficult/Somewhat difficult	0.872	0.468–1.624	0.666
Very difficult/ Can’t do at all	0.709	0.425–1.182	0.187
**Go to events**			
Little difficult/Somewhat difficult	0.697	0.395–1.228	0.212
Very difficult/ Can’t do at all	0.780	0.470–1.296	0.338
**Participate in social activities**			
Little difficult/Somewhat difficult	0.451	0.255–0.795	0.006
Very difficult/ Can’t do at all	0.781	0.462–1.319	0.354
Ref-Not at all category			
